# Poly[μ_6_-(naphthalene-2,6-di­carboxyl­ato)-bis­(aqua­lithium)]

**DOI:** 10.1107/S1600536814013130

**Published:** 2014-07-02

**Authors:** Lionel Fédèle, Frédéric Sauvage, Matthieu Becuwe, Jean-Noël Chotard

**Affiliations:** a Laboratoire de Réactivité et Chimie des Solides (LRCS), Université de Picardie Jules Verne, CNRS UMR 7314, 33 rue Saint Leu, 80039, Amiens, France

## Abstract

The title compound, [Li_2_(C_12_H_6_O_4_)(H_2_O)_2_]_*n*_, crystallizes with one half of the molecular entities in the asymmetric unit. The second half is gererated by inversion symmetry. The crystal structure has a layered arrangement built from distorted edge-sharing LiO_3_(OH)_2_ tetra­hedra parallel to (100), with naphthalenedi­carboxyl­ate bridging the LiO_3_(OH)_2_ layers along the [100] direction. Hydrogen bonding between the water molecule and adjacent carboxylate groups consolidates the packing.

## Related literature   

For the synthesis and crystal structure of 2,6-naphthalenedi­carb­oxy­lic acid, see Kaduk & Golab (1999 ▶). For the synthesis and crystal structure of dilithium-2,6-naphthalene di­carboxyl­ate [Li_2_(2,6-NDC)], see: Banerjee *et al.* (2009*a* ▶). For related compounds, see: Banerjee *et al.* (2009*b* ▶). [Li_2_(2,6-NDC)] was recently reported to exhibit good electrochemical performance, see: Fédèle *et al.* (2014 ▶).
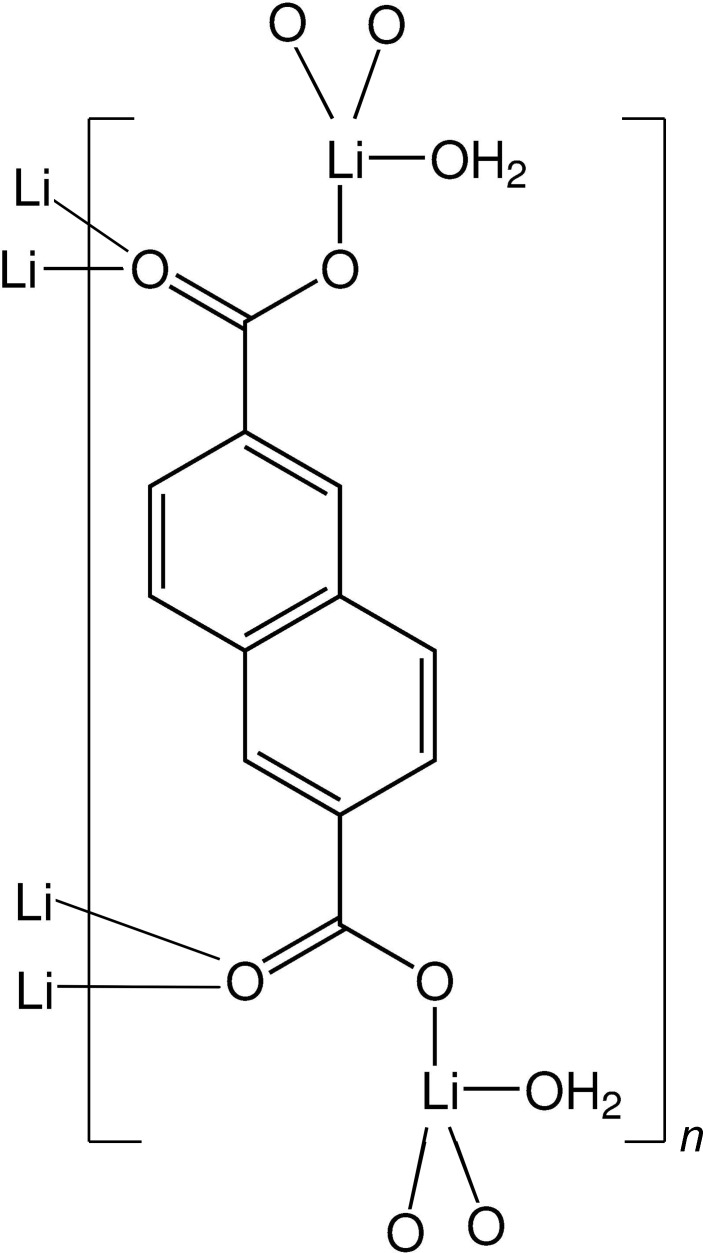


## Experimental   

### 

#### Crystal data  

[Li_2_(C_12_H_6_O_4_)(H_2_O)_2_]
*M* *_r_* = 132.04
Monoclinic, 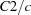

*a* = 23.5695 (18) Å
*b* = 6.8115 (5) Å
*c* = 7.5327 (6) Å
β = 90.325 (3)°
*V* = 1209.31 (16) Å^3^
*Z* = 8
Mo *K*α radiation
μ = 0.11 mm^−1^
*T* = 293 K
0.12 × 0.05 × 0.03 mm

#### Data collection  

Bruker D8 Venture diffractometer
Absorption correction: multi-scan (*SADABS*; Bruker, 2007 ▶) *T*_min_ = 0.707, *T*_max_ = 0.746
12848 measured reflections
1388 independent reflections
1032 reflections with *I* > 2σ(*I*)
*R*_int_ = 0.050

#### Refinement  

*R*[*F* ^2^ > 2σ(*F* ^2^)] = 0.043
*wR*(*F* ^2^) = 0.109
*S* = 1.06
1388 reflections
99 parameters
H atoms treated by a mixture of independent and constrained refinement
Δρ_max_ = 0.23 e Å^−3^
Δρ_min_ = −0.24 e Å^−3^

### 

Data collection: *APEX2* (Bruker, 2007 ▶); cell refinement: *SAINT* (Bruker, 2007 ▶); data reduction: *SAINT*; program(s) used to solve structure: *SHELXS97* (Sheldrick, 2008 ▶); program(s) used to refine structure: *SHELXLE* (Hübschle *et al.*, 2011 ▶); molecular graphics: *VESTA* (Momma & Izumi, 2011 ▶); software used to prepare material for publication: *publCIF* (Westrip, 2010 ▶).

## Supplementary Material

Crystal structure: contains datablock(s) I. DOI: 10.1107/S1600536814013130/pj2011sup1.cif

Structure factors: contains datablock(s) I. DOI: 10.1107/S1600536814013130/pj2011Isup2.hkl

Supporting information file. DOI: 10.1107/S1600536814013130/pj2011Isup3.mol

CCDC reference: 1006973

Additional supporting information:  crystallographic information; 3D view; checkCIF report

## Figures and Tables

**Table 1:** Hydrogen-bond geometry (Å, °)

*D*—H⋯*A*	*D*—H	H⋯*A*	*D*⋯*A*	*D*—H⋯*A*
O2—H1*W*⋯O1^i^	0.81 (3)	2.10 (3)	2.905 (2)	176 (3)
O2—H2*W*⋯O3^ii^	0.89 (3)	2.01 (3)	2.883 (2)	169 (3)

Symmetry codes: (i) 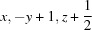; (ii) 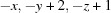.

**Fig. 1.**
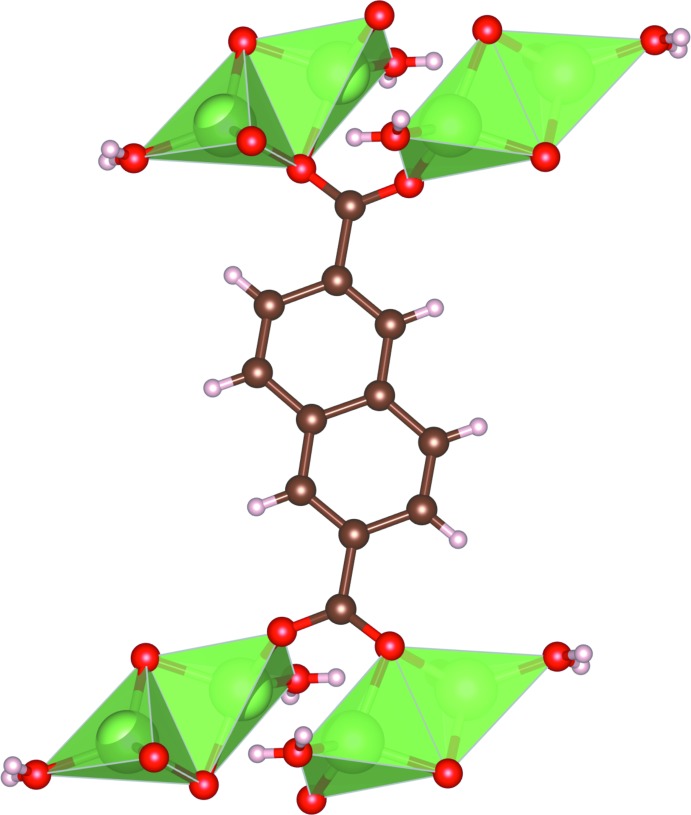
Molecular view of the title compound. Li, O, C, and H atoms are represented by light green, red, brown and white spheres respectively

**Fig. 2.**
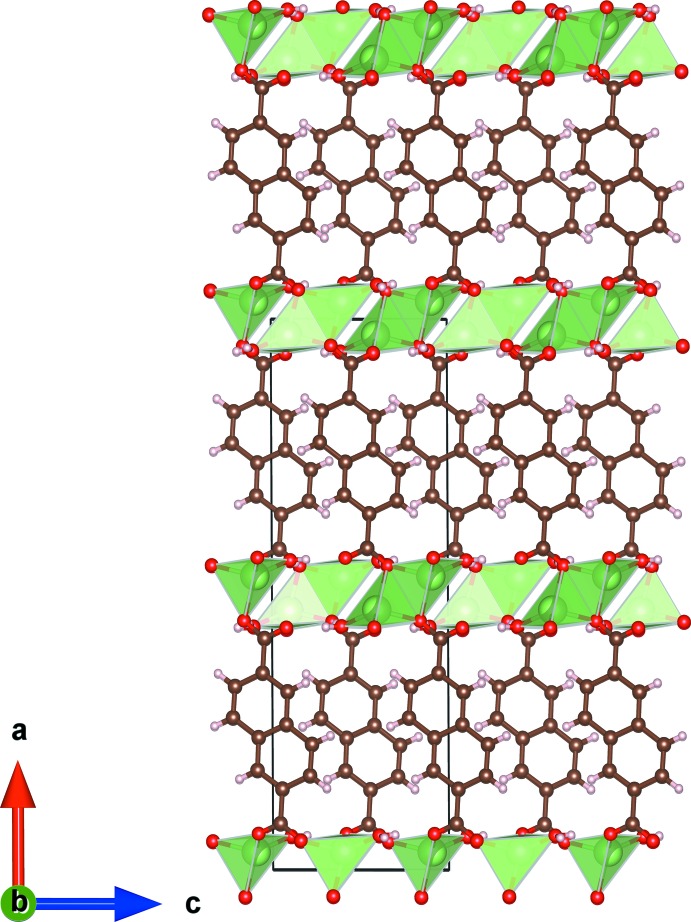
Crystal structure of the title compound view along the b axis. Layers of LiO3(OH2) edge-sharing tetrahedra in the (yz) plane are connected via naphthalene dicarboxylate molecules.

## supplementary crystallographic information

### S1. Comment

For the last 30 years, inorganic compounds involving at least one transition metal as a redox center (such as LiCoO_2_ or LiFePO_4_) have been the traditional electrodes for Li-ion batteries. While they exhibit good performances in terms of cyclability, output voltage capacity, main drawbacks such as toxicity, sustainability and eco-conception still remain. In this context, organic based electrodes for Li-ion batteries recently regained attention. Among them, the dilithium 2,6-naphthalene dicarboxylate (Li_2_-2,6-NDC) was recently reported to exhibit good electrochemical performances (Fédèle *et al.* 2014). The title compound (dilithium 2,6-naphthalene dicarboxylate dihydrate) is the hydrated form of the Li_2_-2,6-NDC. In the former, pairs of edge-sharing LiO_4_ tetrahedra are connected to each other by corners (Banerjee *et al.* 2009*a*). In the hydrated form, the corner sharing arrangement is no longer possible as one oxygen is replaced by a water molecule. Edge-sharing LiO_3_(OH_2_) tetrahedra are connected into sheets that extend in the *yz* plane. These are linked by the naphthalene dicarboxylate into a 3-D array. Crystal data, data collection and structure refinement details are summarized in Table 1.

### S2. Experimental

Reagent and chemicals. The 2,6 naphthalene dicarboxylic acid (98+%) and lithium hydroxide were purchased from Alfa Aesar and Sigma-Aldrich, respectively. They were used as received without further purification. De-ionized water was utilized for the synthesis of the di-lithium salt.

Hydrothermal Lithiation procedure. 1 g of 2,6-naphthalene dicarboxylic acid (4.6 mmol) was added into 10 ml de-ionized water and added to a 23 ml autoclave. Two equivalents of anhydrous lithium hydroxide (222 mg, 9.3 mmol) were incorporated with the naphthalene derivative. The autoclave was then placed into a temperature controlled oven set at 150°C for 12 h duration before to be cooled down to room temperature with a ramp of 10°C/h. The resulting green solution was poured into a 50 ml beaker while the excess water was slowly evaporated under ambient conditions to form the colorless single crystals of the di-lithium-2,6-naphthalene dicarboxylate dihydrate.

### S3. Refinement

All H-atoms were positioned geometrically and refined using a riding model with C—H = 0.90–0.93 Å and with *U*_iso_(H) = 1.2Ueq(C). The H atoms of the aqua ligand (H1W and H2W) were found by Fourier difference map and further refined without any constrains.

### Crystal data

**Table:**

[Li_2_(C_12_H_6_O_4_)(H_2_O)_2_]	*Z* = 8
*M**_r_* = 132.04	*F*(000) = 544
Monoclinic, *C*2/*c*	*D*_x_ = 1.450 Mg m^−^^3^
Hall symbol: -C 2yc	Mo *K*α radiation, λ = 0.71073 Å
*a* = 23.5695 (18) Å	µ = 0.11 mm^−^^1^
*b* = 6.8115 (5) Å	*T* = 293 K
*c* = 7.5327 (6) Å	Prism, colourless
β = 90.325 (3)°	0.12 × 0.05 × 0.03 mm
*V* = 1209.31 (16) Å^3^	

### Data collection

**Table:**

Bruker D8 Venture diffractometer	1388 independent reflections
Radiation source: fine-focus sealed tube	1032 reflections with *I* > 2σ(*I*)
Multilayer optics monochromator	*R*_int_ = 0.050
phi scan	θ_max_ = 27.5°, θ_min_ = 3.1°
Absorption correction: multi-scan (*SADABS*; Bruker, 2007)	*h* = −30→29
*T*_min_ = 0.707, *T*_max_ = 0.746	*k* = −8→8
12848 measured reflections	*l* = −9→9

### Refinement

**Table:**

Refinement on *F*^2^	Primary atom site location: structure-invariant direct methods
Least-squares matrix: full	Secondary atom site location: difference Fourier map
*R*[*F*^2^ > 2σ(*F*^2^)] = 0.043	Hydrogen site location: inferred from neighbouring sites
*wR*(*F*^2^) = 0.109	H atoms treated by a mixture of independent and constrained refinement
*S* = 1.06	*w* = 1/[σ^2^(*F*_o_^2^) + (0.0452*P*)^2^ + 1.0165*P*] where *P* = (*F*_o_^2^ + 2*F*_c_^2^)/3
1388 reflections	(Δ/σ)_max_ = 0.010
99 parameters	Δρ_max_ = 0.23 e Å^−^^3^
0 restraints	Δρ_min_ = −0.24 e Å^−^^3^

### Special details

**Table:**

Geometry. All e.s.d.'s (except the e.s.d. in the dihedral angle between two l.s. planes) are estimated using the full covariance matrix. The cell e.s.d.'s are taken into account individually in the estimation of e.s.d.'s in distances, angles and torsion angles; correlations between e.s.d.'s in cell parameters are only used when they are defined by crystal symmetry. An approximate (isotropic) treatment of cell e.s.d.'s is used for estimating e.s.d.'s involving l.s. planes.
Refinement. Refinement of *F*^2^ against ALL reflections. The weighted *R*-factor *wR* and goodness of fit *S* are based on *F*^2^, conventional *R*-factors *R* are based on *F*, with *F* set to zero for negative *F*^2^. The threshold expression of *F*^2^ > σ(*F*^2^) is used only for calculating *R*-factors(gt) *etc*. and is not relevant to the choice of reflections for refinement. *R*-factors based on *F*^2^ are statistically about twice as large as those based on *F*, and *R*- factors based on ALL data will be even larger·A single gross outlier (reflection 3 3 3)was omitted from the final refinement.

### Fractional atomic coordinates and isotropic or equivalent isotropic displacement parameters (Å^2^)

**Table:**

	*x*	*y*	*z*	*U*_iso_*/*U*_eq_	
O1	0.06322 (5)	0.42265 (18)	0.41860 (17)	0.0357 (3)	
H1W	0.0611 (12)	0.761 (5)	0.695 (4)	0.082 (10)*	
H2W	0.0553 (11)	0.949 (5)	0.635 (4)	0.089 (10)*	
O2	0.05985 (7)	0.8245 (3)	0.6045 (2)	0.0496 (4)	
O3	−0.05040 (4)	0.75789 (19)	0.34106 (15)	0.0323 (3)	
C1	0.08206 (6)	0.3193 (2)	0.5432 (2)	0.0252 (4)	
C2	0.14481 (6)	0.2809 (2)	0.5548 (2)	0.0251 (4)	
C3	0.17876 (6)	0.3237 (2)	0.4137 (2)	0.0261 (4)	
H3	0.1627	0.3764	0.3112	0.031*	
C4	0.23825 (6)	0.2893 (2)	0.4209 (2)	0.0245 (4)	
C5	0.22574 (7)	0.1697 (3)	0.7239 (2)	0.0300 (4)	
H4	0.2412	0.1206	0.8288	0.036*	
C6	0.16892 (7)	0.2010 (3)	0.7120 (2)	0.0303 (4)	
H5	0.1457	0.1700	0.8074	0.036*	
Li1	0.03075 (12)	0.6849 (4)	0.4040 (4)	0.0305 (6)	

### Atomic displacement parameters (Å^2^)

**Table:**

	*U*^11^	*U*^22^	*U*^33^	*U*^12^	*U*^13^	*U*^23^
O1	0.0262 (6)	0.0378 (7)	0.0432 (7)	0.0097 (5)	−0.0039 (5)	0.0041 (6)
O2	0.0714 (11)	0.0404 (9)	0.0367 (8)	−0.0102 (8)	−0.0102 (7)	−0.0003 (7)
O3	0.0197 (5)	0.0452 (7)	0.0322 (6)	−0.0007 (5)	0.0075 (5)	−0.0052 (6)
C1	0.0190 (7)	0.0253 (8)	0.0313 (9)	0.0018 (6)	0.0016 (6)	−0.0084 (7)
C2	0.0180 (7)	0.0241 (8)	0.0333 (9)	0.0019 (6)	0.0022 (6)	−0.0009 (7)
C3	0.0208 (8)	0.0285 (8)	0.0290 (8)	0.0037 (7)	−0.0011 (6)	0.0035 (7)
C4	0.0215 (8)	0.0236 (8)	0.0283 (8)	0.0021 (6)	0.0010 (6)	0.0023 (7)
C5	0.0241 (8)	0.0365 (9)	0.0293 (9)	0.0035 (7)	0.0005 (6)	0.0096 (8)
C6	0.0239 (8)	0.0366 (9)	0.0304 (9)	0.0016 (7)	0.0063 (6)	0.0053 (7)
Li1	0.0275 (14)	0.0337 (15)	0.0302 (14)	0.0016 (12)	−0.0007 (11)	0.0021 (12)

### Geometric parameters (Å, º)

**Table:**

O1—C1	1.253 (2)	C3—C4	1.422 (2)
O1—Li1	1.946 (3)	C3—H3	0.9300
O2—Li1	1.910 (3)	C4—C5^iii^	1.414 (2)
O2—H1W	0.81 (3)	C4—C4^iii^	1.417 (3)
O2—H2W	0.89 (3)	C5—C6	1.359 (2)
O3—C1^i^	1.265 (2)	C5—C4^iii^	1.414 (2)
O3—Li1^ii^	1.969 (3)	C5—H4	0.9300
O3—Li1	2.030 (3)	C6—H5	0.9300
C1—O3^i^	1.265 (2)	Li1—O3^ii^	1.969 (3)
C1—C2	1.504 (2)	Li1—C1^i^	2.691 (3)
C1—Li1^i^	2.691 (3)	Li1—Li1^ii^	2.728 (6)
C2—C3	1.365 (2)	Li1—Li1^i^	3.250 (6)
C2—C6	1.419 (2)		
			
C1—O1—Li1	134.09 (14)	C5—C6—C2	120.35 (15)
Li1—O2—H1W	114 (2)	C5—C6—H5	119.8
Li1—O2—H2W	129.8 (19)	C2—C6—H5	119.8
H1W—O2—H2W	107 (3)	O2—Li1—O1	105.84 (15)
C1^i^—O3—Li1^ii^	133.11 (14)	O2—Li1—O3^ii^	122.04 (16)
C1^i^—O3—Li1	107.22 (13)	O1—Li1—O3^ii^	100.98 (14)
Li1^ii^—O3—Li1	86.02 (13)	O2—Li1—O3	113.35 (15)
O1—C1—O3^i^	122.81 (14)	O1—Li1—O3	127.43 (16)
O1—C1—C2	119.05 (14)	O3^ii^—Li1—O3	86.88 (12)
O3^i^—C1—C2	118.13 (14)	O2—Li1—C1^i^	103.83 (13)
O1—C1—Li1^i^	76.73 (11)	O1—Li1—C1^i^	111.74 (13)
O3^i^—C1—Li1^i^	46.09 (10)	O3^ii^—Li1—C1^i^	112.28 (13)
C2—C1—Li1^i^	164.10 (13)	O3—Li1—C1^i^	26.69 (6)
C3—C2—C6	119.84 (14)	O2—Li1—Li1^ii^	149.25 (11)
C3—C2—C1	119.89 (14)	O1—Li1—Li1^ii^	104.78 (10)
C6—C2—C1	120.27 (14)	O3^ii^—Li1—Li1^ii^	47.92 (9)
C2—C3—C4	121.15 (15)	O3—Li1—Li1^ii^	46.06 (9)
C2—C3—H3	119.4	C1^i^—Li1—Li1^ii^	66.74 (11)
C4—C3—H3	119.4	O2—Li1—Li1^i^	101.12 (15)
C5^iii^—C4—C4^iii^	119.35 (17)	O1—Li1—Li1^i^	55.92 (10)
C5^iii^—C4—C3	122.30 (14)	O3^ii^—Li1—Li1^i^	136.12 (18)
C4^iii^—C4—C3	118.35 (17)	O3—Li1—Li1^i^	82.63 (12)
C6—C5—C4^iii^	120.93 (15)	C1^i^—Li1—Li1^i^	58.82 (9)
C6—C5—H4	119.5	Li1^ii^—Li1—Li1^i^	98.18 (13)
C4^iii^—C5—H4	119.5		
			
Li1—O1—C1—O3^i^	71.7 (2)	C1—O1—Li1—O2	24.8 (2)
Li1—O1—C1—C2	−109.69 (19)	C1—O1—Li1—O3^ii^	152.87 (15)
Li1—O1—C1—Li1^i^	72.46 (19)	C1—O1—Li1—O3	−112.6 (2)
O1—C1—C2—C3	−13.1 (2)	C1—O1—Li1—C1^i^	−87.6 (2)
O3^i^—C1—C2—C3	165.63 (15)	C1—O1—Li1—Li1^ii^	−158.04 (16)
Li1^i^—C1—C2—C3	159.3 (4)	C1—O1—Li1—Li1^i^	−68.08 (18)
O1—C1—C2—C6	166.67 (15)	C1^i^—O3—Li1—O2	−73.79 (18)
O3^i^—C1—C2—C6	−14.6 (2)	Li1^ii^—O3—Li1—O2	152.12 (13)
Li1^i^—C1—C2—C6	−21.0 (5)	C1^i^—O3—Li1—O1	61.0 (2)
C6—C2—C3—C4	0.2 (3)	Li1^ii^—O3—Li1—O1	−73.05 (18)
C1—C2—C3—C4	179.88 (14)	C1^i^—O3—Li1—O3^ii^	162.48 (11)
C2—C3—C4—C5^iii^	179.28 (16)	Li1^ii^—O3—Li1—O3^ii^	28.40 (17)
C2—C3—C4—C4^iii^	−0.5 (3)	Li1^ii^—O3—Li1—C1^i^	−134.08 (16)
C4^iii^—C5—C6—C2	−1.7 (3)	C1^i^—O3—Li1—Li1^ii^	134.08 (16)
C3—C2—C6—C5	1.0 (3)	C1^i^—O3—Li1—Li1^i^	25.18 (15)
C1—C2—C6—C5	−178.74 (15)	Li1^ii^—O3—Li1—Li1^i^	−108.90 (8)

Symmetry codes: (i) −*x*, −*y*+1, −*z*+1; (ii) −*x*, *y*, −*z*+1/2; (iii) −*x*+1/2, −*y*+1/2, −*z*+1.

### Hydrogen-bond geometry (Å, º)

**Table:**

*D*—H···*A*	*D*—H	H···*A*	*D*···*A*	*D*—H···*A*
O2—H1*W*···O1^iv^	0.81 (3)	2.10 (3)	2.905 (2)	176 (3)
O2—H2*W*···O3^v^	0.89 (3)	2.01 (3)	2.883 (2)	169 (3)

Symmetry codes: (iv) *x*, −*y*+1, *z*+1/2; (v) −*x*, −*y*+2, −*z*+1.
